# New Approaches for Enhanced Detection of Enteroviruses from Hawaiian Environmental Waters

**DOI:** 10.1371/journal.pone.0032442

**Published:** 2012-05-02

**Authors:** Christina Connell, Hsin-I Tong, Zi Wang, Erin Allmann, Yuanan Lu

**Affiliations:** Departments of Public Health Sciences and Microbiology, University of Hawaii at Manoa, Honolulu, Hawaii, United States of America; University of Hyderabad, India

## Abstract

Health risks associated with sewage-contaminated recreational waters are of important public health concern. Reliable water monitoring systems are therefore crucial. Current recreational water quality criteria rely predominantly on the enumeration of bacterial indicators, while potentially dangerous viral pathogens often remain undetected. Human enteric viruses have been proposed as alternative indicators; however, their detection is often hindered by low viral concentrations present in the environment. Reported here are novel and effective laboratory protocols for viral concentration and highly sensitive and optimized RT-PCR for the efficient detection of enteroviruses, an important enteric virus subset, in Hawaiian environmental waters. Eighteen published enterovirus primer pairs were comparatively evaluated for detection sensitivity. The primer set exhibiting the lowest detection limit under optimized conditions, EQ-1/EQ-2, was validated in a field survey of 22 recreational bodies of water located around the island of Oahu, Hawaii. Eleven sites tested positive for enterovirus, indicating fecal contamination at these locations. As an additional means of viral concentration, shellfish were collected from 9 sample sites and subjected to dissection, RNA extraction, and subsequent RT-PCR. Shellfish tissue from 6 of 9 sites tested positive for enterovirus. The techniques implemented here are valuable resources to aid accurate reflection of microbial contamination in Hawaii’s environmental waters.

## Introduction

Sewage-contaminated recreational water can pose numerous health risks to the public; effective water quality monitoring is therefore absolutely essential [1]. Currently, microbiological water quality is primarily assessed via bacterial indicators such as enterococci, fecal coliform, and total coliform bacteria. However, these indicators often fail to reflect the presence of important hazardous viruses [2]. This is of important concern, as viral pathogens shed in human feces may compromise public safety by polluting recreational waters that meet bacterial indicator standards. Additionally, these bacterial indicators may grow naturally in tropical environments, resulting in inaccurate assessment of water pollution levels [3]. Therefore, alternative monitoring systems are needed to improve the surveillance of recreational waters and secure public protection from waterborne disease [4].

Human enteric viruses, represented by the astroviruses, rotaviruses, noroviruses, adenoviruses, and picornaviruses, have been associated with many waterborne outbreaks and are suggested as alternative indicators of microbial water quality [5], [6]. Enteric viruses are primarily transmitted via the fecal-oral route, and viral particles are shed in extremely high numbers from infected individuals [6]. Although most enteric virus infections are primarily associated with diarrhea and self-limiting gastroenteritis, they may also cause hepatitis, conjunctivitis, and respiratory infections. Additionally, in immunocompromised persons, enteric viruses have been associated with aseptic meningitis, encephalitis, and paralysis, all of which have high mortality rates [6]. Common wastewater treatment processes fail to completely inactivate these viruses [7], rendering recreational waters in areas such as Hawaii, where primary-treated sewage is discharged into the sea on a normal basis, vulnerable to viral contamination. Additionally, enteric viruses are able to survive in the environment under a wide pH range and for extended time periods [8]. Due to large viral loads released into sewage-impacted waters, increased environmental persistence compared to indicator bacteria, and the significant role viruses play in waterborne disease, enteric viruses show promising potential to be used as alternative indicators for a more accurate depiction of recreational water quality [6]. This is especially significant in the state of Hawaii, where residents and tourists alike enjoy year-round recreational activities in the local waters.

Although the utilization of enteric viruses as alternative water quality indicators is desirable, conventional methods for viral isolation from water are laborious, time-consuming, and inefficient [9]. A major problem encountered is the effective detection of low levels of viruses present in large bodies of water [10]. Because enteric viruses are able to establish infection in humans at low infectious doses, extremely sensitive detection assays are needed. The polymerase chain reaction (PCR) has become an invaluable resource for environmental virologists, favored for its rapidity, sensitivity, specificity, and relative ease-of-use. However, the presence of inhibiting compounds, which can lead to false-negative results, presents an additional barrier [9]–[11]. Detection challenges may be overcome by improved methods for viral concentration from water samples and by efficient inhibitor removal during nucleic acid extraction [11].

Here, we have developed a highly optimized molecular protocol for the effective detection of enteroviruses (EnV) from Hawaiian environmental waters. Enteroviruses, RNA viruses belonging to the Picornavirus family and consisting of coxsackievirus, poliovirus, echovirus, and the numbered enteroviruses, are the most commonly detected enteric viruses in polluted waters and are estimated to cause 30 – 50 million infections in the US annually [12], [13]. The EnV disease spectrum is wide, including gastroenteritis, respiratory infection, diabetes, heart disease, bronchiolitis, conjunctivitis, meningitis, paralysis, and the common cold [6]. Because these viruses are common, fecally shed in extremely high numbers from infected individuals, highly tolerant to salinity and temperature fluctuations, and stable in the environment for extended time periods [8], they have been suggested as a parameter for evaluating viral pollution of environmental waters [13], [14]. The availability of permissive cell lines for determining EnV infectivity greatly enhances the attractiveness of using this important enteric virus subset as an alternative indicator of water quality [6].

Additionally, in order to enhance viral concentration from environmental water samples, we briefly report the potential utilization of marine bivalves as bioindicators of water quality. Because these animals are filter feeders, they process large volumes of water daily, which causes viruses to accumulate within their tissues at a concentration higher than that in the surrounding water [15], [16]. Combining this natural bioconcentration phenomenon with our highly optimized RT-PCR protocol for EnV detection shows promising potential to aid in efficient surveillance of Hawaiian environmental waters.

## Materials and Methods

### Wastewater Sample Collection

Because multiple enteroviral strains are fecally shed in high loads from infected individuals [14], urban wastewater was used as the nucleic acid source for optimization of EnV molecular amplification. Wastewater was obtained from the Sand Island Wastewater Treatment Plant, responsible for processing approximately 85% of Oahu’s wastewater. This facility utilizes an advanced primary treatment, disinfecting sewage via ultraviolet (UV) radiation before releasing it 1.7 miles offshore into the ocean [17]. Samples were collected in 2-L sterile, polypropylene containers from the following three treatment stages: raw influent, post-primary clarification/pre-UV disinfection, and post-UV disinfection/effluent. Samples were transported on ice to a BSL-2 laboratory and processed immediately.

### Environmental Water Sample Collection

**Figure 1:**
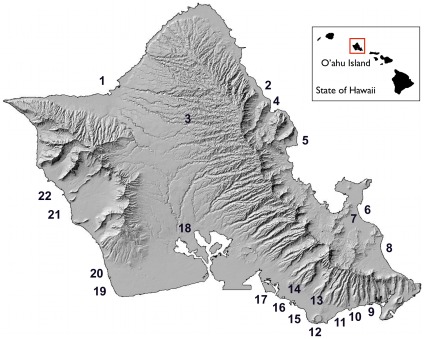
Environmental sampling sites around the island of O’ahu, Hawaii.

Between June 2010 and October 2011, twenty-two surface water samples were collected from various marine and freshwater sites around the island of Oahu (Figure 1). No specific permits were required for sample collection. Marine sites include Sand Island State Recreational Area, Kailua Bay, Waikiki Beach, Pokai Bay, Maunalua Bay, Kualoa Regional Park, West Loch Community Shoreline Park, Kahala Beach, and the beach parks of Ala Moana, Diamond Head, Maili, Waialae, Kaiaka Bay, Kahana Bay, Ko Olina (Lagoons 3 and 4), Bellows Field, and Punalu’u. Freshwater sites include Wahiawa Reservoir, Manoa Stream, and Kaelepulu Stream. The sample collected from Ala Wai Canal was brackish. All sampling locations receive, to varying degrees, considerable recreational activity, including swimming, snorkeling, surfing, kayaking, canoeing, boating, and fishing. 2-L samples were collected in sterile, polypropylene containers and transported on ice to the laboratory for immediate processing. A 2-L field blank consisting of double-distilled H_2_O was prepared as a negative control. A positive control was prepared by spiking 2-L of seawater from Diamond Head Beach Park with 100 ml EnV-positive wastewater influent.

### Sample Concentration, Nucleic Acid Extraction, and RT-PCR

Sewage and environmental samples were processed using a filtration-based method described previously by Tong and Lu, 2011 [17]. In order to aid in viral absorption, MgCl_2_ solution was mixed into sewage and freshwater samples prior to filtration at a final concentration of 25 mM. 100 mL of sewage and 2 L of environmental water samples were filtered through 0.45-µM pore size, type HA membranes (Millipore Corporation, MA) on a filtration manifold under vacuum. (It should be noted that for three water samples with high sediment content, collected from Bellows Field Beach Park, West Loch Community Shoreline Park, and Kaelepulu Stream, filters became clogged before 2 L were able to pass; therefore, smaller volumes of 0.80 L, 0.80 L, and 0.50 L were passed, respectively.) Nucleic acids were extracted from the recovered membranes using the PowerWater RNA Isolation Kit, supplied by MoBio Laboratories, CA, according to a modified protocol designed for separate extraction of both RNA and DNA, described previously by Tong, 2011 [37]. Seven microliters of RNA extracted from each sample were used as template for RT-PCR, performed with the DyNAmo cDNA synthesis kit (New England Biolabs, NEB, MA) according to the manufacturer’s instructions. Random hexamers were used as primers.

### Comparative Analysis of Published Enterovirus Primer Sets

While several RT-PCR protocols have already been established for the detection of EnV [18]–[31], little is known about their comparative detection sensitivities, which is of utmost importance when assessing microbial water quality. Therefore, eighteen published primer sets, specific for amplifying various regions of the EnV genome, were selected in this study in a comparative evaluation of detection sensitivity (Table 1). The primer sets chosen are specific for all pathogenic but highly diverse human enteroviruses, with the exception of EvVP1F/EvVP1R, which specifically selects for EV71, causative agent of hand, foot, and mouth disease in children [20]. All primer sets were initially tested under standard PCR conditions using single-source cDNA from wastewater influent as the nucleic acid template. Five microliters of cDNA was added to 20 µL PCR mix containing 1X *Taq* reaction buffer (NEB, MA), 2.0 mM MgCl_2_ solution (NEB, MA), 200 nM of each dNTP (Sigma-Adrich, MO), 400 nM of forward and reverse primers (Integrated DNA Technologies, IA), and 2 units of *Taq* DNA polymerase (provided by Dr. Tung Hoang, University of Hawaii at Manoa). Reaction tubes were placed in a Mastercycler® Gradient (Eppendorf, Germany) for an initial denaturation at 94°C for 5 min., followed by 40 cycles of denaturation at 94°C for 30 sec., annealing at 56°C for 20 sec., and extension at 72°C for 30 sec., completed by a final extension at 72°C for 5 min. EnV detection was analyzed by gel electrophoresis. 10 µL PCR product+2 µL 6x loading dye was loaded into the wells of an ethidium-bromide stained 2% agarose gel in 0.5x TBE buffer, to which 120V was applied until sufficient fragment migration had occurred. A 50-bp DNA ladder (NEB, MA) was used for indication of PCR product fragment size. The Molecular Imager Gel Doc XR+system (BioRad Laboratories, Inc., CA) was used to visualize results under UV light.

**Table 1:** Enterovirus primer sets employed in comparative analysis.

Primer	Sequence (5'–3')	Amplicon size (bp)	Ref.
EV1/EV2	CGGCCCCTGAATGCGGC/CACCGGATGGCCAATCCA	196	18
EntAF/R	TNCARGCWGCNGARACNGG/ANGGRTTNGTNGMWGTYTGCCA	414	
EntBF/R	GCNGYNGARACNGGNCACAC/CTNGGRTTNGTNGANGWYTGCC	397	
EntCF/R	TNACNGCNGTNGANACHGG/TGCCANGTRTANTCRTCCC	395	
EQ-1/EQ-2	ACATGGTGTGAAGAGTCTATTGAGCT/CCAAAGTAGTCGGTTCCGC	142	19
EvVP1F/R	GAGAGTTCTATAGGGGACAGT/AGCTGTGCTATGTGAATTAGGAA	204	20
2AB/2C3A	GAIGYIATGGARCARGG/GGICCYTGRAAIARIGCYTC	1200	21
EV.1/EV.2	GGCCCCTGAATGCGGCTAAT/CAATTGTCACCATAAGCAGCCA	54	22
Lees3/4	CATTCAGGGGCCGGAGGA/AAGCACTTCTGTTTCC	256	23
P1/P3	CAAGCACTTCTGTTTCCCCGG/ATTGTCACCATAAGCAGCCA	440	24
P2/P3	TCCTCCGGCCCCTGAATGCG/ATTGTCACCATAAGCAGCCA	155	
EV-L/-R	CCTCCGGCCCCTGAATG/ACCGCGATGGCCAATCCAA	197	25
Abba1/2	TGTCACCATAAGCAGCC/TCCGGCCCCTGAATGCGGCT	149	26
EVZ1/Z2	CAAGCACTTCTGTTTCCCCGG/ACCCATAGTAGTCGGTTCCGC	388	27
EVF/EVR	CCTGAATGCGGCTAATCC/ATTGTCACCATAAGCAGCCA	144	28
ev1q/ev2q	GATTGTCACCATAAGCAGC/CCCCTGAATGCGGCTAATC	146	29
Ent1/Ent2	CGGGTACCTTTGTACGCCTGT/ATTGTCACCATAAGCAGCCA	534	30
EvUp/Dwn	TGTCACCATAAGCAGCC/TCCGGCCCCTGAATGCGGCT	149	31

PCR conditions for all primer sets that successfully detected EnV from untreated wastewater were then adjusted for optimal sensitivity in preparation for environmental detection. Optimization brackets included annealing temperature, MgCl_2_ concentration, primer concentration, and the presence or absence of 0.1 µg/µL molecular biology grade, protease/nuclease-free, fraction V BSA (NEB, MA) (Table 2). Using the final optimized conditions, primer set detection limits were determined by PCR using 10-fold serial dilutions of influent sewage cDNA template. Detection limits were denoted by the highest dilution yielding a clear, positive detection signal, visualized by performing gel electrophoresis after PCR amplification.

**Table 2:** PCR Condition brackets included in optimization assay.

Condition	Test Range
1. T_anneal_	50–60°C^a^, 2° increments
2. [MgCl_2_]	1.5, 2.0, 3.0, 4.0 mM
3. [Primer]	200, 400, 600, 800, 1000 nM
4. BSA	Presence/Absence (0.1 µg/µL)

a 40-50°C was included if reported T_anneal_ in literature was <50°C.

### Validation through Screening of Sewage and Environmental Waters

The primer set exhibiting the highest sensitivity, EQ-1/EQ-2, was confirmed using cDNA obtained from the three sewage stages described earlier (raw influent, post-clarification/pre-UV disinfection, and post-UV disinfection/effluent). This set was selected as the optimal candidate for surveillance of EnV presence in the environment; the twenty-two environmental water samples were then tested for EnV contamination using the newly-optimized PCR conditions (see Table 3).

**Table 3:** Optimized amplification conditions and detection limits of seven successful primer sets.

Primer Set	T_anneal_	[MgCl_2_]	[Primer]	BSA	Detection Limit^a^
EQ-1/EQ-2	58–60°C	1.5 mM	600 nM	+	10^–7^ X
P1/P3	48°C	3.0 mM	800 nM	+	10^–4^ X
P2/P3	48°C	1.5 mM	400 nM	+	10^–4^ X
EV-L/-R	55–58°C	3.0 mM	400 nM	+	10^–6^ X
EVZ1/Z2	56°C	2.0 mM	1 µM	+	10^–4^ X
EVF/EVR	58–60°C	3.0 mM	1 µM	+	10^–5^ X
ev1q/ev2q	58–60°C	1.5 mM	800 nM	+	10^–6–7^ X

a As determined by lowest 10X serial dilution of wastewater influent cDNA template yielding positive EnV detection, visualized by performing gel electrophoresis after PCR amplification.

### Shellfish as Potential Indicators of Water Quality

From nine of the beaches where water samples were obtained, marine bivalves *Isognomon spp.* were collected from reef crevices and from underneath rocks. Between 18 and 55 specimens were collected from each site, depending on size and availability. No specific permits were required for specimen collection. Following transport to the laboratory on ice, shellfish were immediately shucked, and nucleic acids were extracted from internal digestive tissues in 1.0–2.0 g aliquots using the MoBio PowerSoil RNA Isolation Kit+DNA Elution Accessory Kit (MoBio Laboratories, CA), according to the manufacturer’s instructions. Extracted RNA was DNase-trested using the RTS DNase Kit (MoBio Laboratories, CA), according to the manufacturer’s instructions. Nucleic acids were stored at –80°C. RNA was subjected to RT-PCR using the previously described optimized conditions in order to test for the presence of EnV; results were visualized by performing gel electrophoresis as described above.

### E. Coli Amplification as Internal Control

It is well known that environmental water and shellfish samples contain high levels of inhibitory compounds that, if inefficiently removed during sampling processing, can negatively affect downstream molecular analysis [9]–[11]. In order to assess nucleic acid extraction efficiency and inhibitor removal during sample processing, DNA extracted from all water and shellfish samples was tested for the presence of *E. coli*, which is known to grow naturally in the Hawaiian environment and is expected to be readily detectable in all samples [32]. In each 25 µL reaction, 3 µL sample DNA were added to 22 µL PCR mixture containing 1X *Taq* (Mg^2+^ free) reaction buffer, 2.5 mM MgCl_2_ solution, 200 nM dNTP mixture, 0.1 µg/µL BSA, 400 nM of each primer (URL301: TGTTACGTCCTGTAGAAAGCCC, URR-432: AAAACTGCCTGGCACAGCAATT) [33], and 2 units of *Taq* polymerase. The amplification cycle consisted of an initial 5 min. denaturation at 94°C, followed by 35 cycles of 30 sec. denaturation at 94°C, 30 sec. annealing at 60°C, and 30 sec. extension at 72°C, completed by a final 5 min. extension at 72°C. Gel electrophoresis was performed as described above.

### PCR Product Sequencing and Analysis

In order to confirm true EnV detection and identify enteroviral strains present in the Hawaiian environment, selected positive DNA fragments amplified by primer set EQ-1/EQ-2 from sewage, water, and shellfish samples were subjected to DNA sequencing. DNA bands were excised from the 2% agarose gel and recovered using the QIAquick Gel Extraction kit (Qiagen, CA), according to the manufacturer’s instructions. Recovered DNA samples from sewage and water were eluted using 30 µL EB buffer and cloned into pCR®2.1-TOPO® vectors using the TOPO TA Cloning® kit (Invitrogen, CA) according to the manufacturer’s instructions. 8 positive clones from a single influent sewage sample and 5 environmental clones from 5 positive sampling sites (Manoa Stream, Pokai Bay, Kaiaka Beach Park, Waikiki Beach, and Wahiawa Reservoir) were submitted with the M13 forward primer, provided by the commercial kit, to the College of Natural Sciences Advanced Studies of Genomics, Proteomics and Bioinformatics (ASGPB, University of Hawaii at Manoa) for DNA sequencing. Recovered enteric viral DNA amplified from shellfish collected at 3 sampling sites (Waialae Beach Park, Punalu’u Beach Park, Kualoa Regional Park) was submitted for direct sequencing to the same facility. Resulting genomic sequences were aligned and compared with all available EnV sequences listed in the National Center for Biotechnology Information (NCBI) databank using the Basic Local Alignment Search Tool (BLAST).

### Infectivity Assay

Because positive detection of enterovirus by PCR amplification does not necessarily correlate with the presence of viable and infectious viruses [3], an initial infectivity assay was performed by infecting buffalo green monkey kidney (BGMK) and A549 cell lines with viruses isolated from EnV-positive wastewater influent. Both of these cell lines are commonly used for the isolation of waterborne EnV [34], [35]. Isolates were obtained by passing 100 ml sewage sample mixed with 25 mM solution through a filter membrane (as described earlier for subsequent nucleic acid extraction), washing the membrane with 200 ml of 0.5 mM H_2_SO_4_ (pH 3.0) to remove cations, and rinsing the membrane with 10 ml of 1 mM NaOH (pH 10.8) to elute viruses into a flask containing 1 ml 10x TE buffer (pH 8.0) and 40 µl of 100 mM H_2_SO_4_ for immediate neutralization. Eluent to be used to infect A549 cells was concentrated to 0.5 ml using a Pierce® concentrator (20 ml/20 K, Pierce, IL) and combined with 0.5 ml 2X DMEM (Sigma-Aldrich, MO). Eluent to be used to infect BGMK cells was supplemented with AIM (1∶4; 1000 U/1000 ug/2 mM of P/S/G, 25 ug Amp B, 500 ug Gentamicin, with 1x high glucose DMEM) for 2 hours prior to cell infection. BGMK **(**gift from Dr. Philip C Loh) and A549 cell monolayers (ATCC® #CCL-185™, Manassas, VA) were infected at 1∶10, 1∶100, and 1∶1000 dilution rates and grown in T-75cm^2^ culture flasks in a humidified 5.0% CO_2_ incubator set at 37°C. Cells were grown in Minimum essential medium (MEM) (BGMK) and high glucose DMEM (A549) and supplemented with 1% antibiotics (Sigma-Aldrich, MO) and 10% heat-inactivated fetal bovine serum (FBS, HyClone, UT). Cells were passaged via trypsinization and split at a 1∶3 ratio every 2–3 days. Cells were routinely examined for the appearance of any viral-induced cytopathic effect (CPE).

### Biostatistical Analysis

A score test was performed to examine the association between the two EnV detection methods (coastal water samples vs. marine shellfish).

## Results

### RT-PCR Condition Optimization and Detection Sensitivity

Of the initial 18 primer sets tested, only 7 generated PCR products of the expected size from untreated sewage, indicating positive EnV detection (EQ-1/EQ-2, Primer 1/Primer 3, Primer 2/Primer 3, EV-L/EV-R, EVZ1/EVZ2, EVF/EVR, ev1qia/ev2qia). Conditions for these 7 pairs were then optimized for their use in conventional PCR. Optimal annealing temperatures, salt concentrations, primer concentrations, and BSA presence/absence for these 7 primer sets, along with their resulting detection limits, are summarized in Table 3. It was found that the addition of BSA increased detection strength of all 7 sets of primer pairs. Detection limits significantly varied among these primer sets, differing by as much as 1000-fold. The primer set exhibiting the highest sensitivity, EQ-1/EQ-2, with a detection limit of 10^–7^ X, was selected for further experimentation. This primer set generates a 142 base pair amplicon within the highly conserved 5′ UTR region of the EnV genome, including parts of domains IV and V of the internal ribosomal entry site [19].

### Enterovirus Detection in Sewage and Environmental Waters

**Figure 2:**
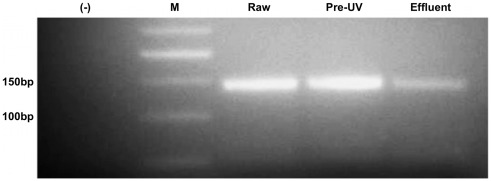
Agarose gel depicting enterovirus detection from urban sewage. Amplified with primer set EQ-1/EQ-2. Detection from 100 mL of raw influent, post-primary clarification/pre-UV disinfection, and post-disinfection/effluent treatment stages. M = 50 bp DNA ladder. (-) = no template control.

Primer set EQ-1/EQ-2′s optimized PCR conditions were confirmed using urban wastewater, resulting in DNA bands of the expected size (142 bp) at all three treatment stages tested (Figure 2). Environmental screening followed, indicating that eleven of the twenty-two sample sites contained EnV contamination, including Diamond Head Beach Park, Pokai Bay, Kailua Bay, Waikiki Beach, Kaiaka Bay Beach Park, Wahiawa Reservoir, Manoa Stream, Ala Moana Beach Park, Ko Olina Beach Park Lagoon 3, Kahala Beach, and Punalu’u Beach Park (Table 4).

**Table 4:** Enterovirus detection in Hawaiian environmental waters.

Map#^a^	Site	Condition	EnVdetection
1	Kaiaka Bay Beach Park	Seawater	+
2	Punalu’u Beach Park	Seawater	+
3	Wahiawa Reservoir	Freshwater	+
4	Kahana Bay Beach Park	Seawater	−
5	Kualoa Regional Park	Seawater	−
6	Kailua Bay	Seawater	+
7	Kaelepulu Stream	Freshwater	−
8	Bellows Field Beach Park	Seawater	−
9	Maunalua Bay	Seawater	−
10	Waialae Beach Park	Seawater	−
11	Kahala Beach	Seawater	+
12	Diamond Head Beach Park	Seawater	+
13	Manoa Stream	Freshwater	+
14	Ala Wai Canal	Brackish	−
15	Waikiki Beach	Seawater	+
16	Ala Moana Beach Park	Seawater	+
17	Sand Island State Recreational Area	Seawater	−
18	West Loch Shoreline Park	Seawater	−
19	Ko Olina Beach Park Lagoon 4	Seawater	−
20	Ko Olina Beach Park Lagoon 3	Seawater	+
21	Maili Beach Park	Seawater	−
22	Pokai Bay	Seawater	+
	Field Blank	ddH_2_O	−
	Spike control	Seawater+sewage	+

a See Figure 1.

### Enterovirus Detection in Shellfish Tissue

Enterovirus was detected in shellfish tissue from six of nine beach sites tested, including Kahala Beach, Kualoa Regional Park, and the beach parks located at Ala Moana, Waialae, Ko Olina Lagoon 3, and Punalu’u. More detailed detection data and a comparison with EnV detection in water samples from corresponding locations is shown in Table 5.

**Table 5:** Enterovirus detection in water vs. shellfish at 9 sampling locations.

Site	EnV detection	
	Water	Shellfish# (+) of Total # Tested
Ala Moana Beach Park	+	12 of 28
Kahala Beach	+	18 of 30
Kahana Bay Beach Park	−	0 of 40
Ko Olina Beach Park Lagoon 3	+	18 of 18
Ko Olina Beach Park Lagoon 4	−	0 of 48
Kualoa Regional Park	−	44 of 55
Punalu’u Beach Park	+	40 of 48
Sand Island State Recreational Area	−	0 of 26
Waialae Beach Park	−	8 of 36

### E. Coli Detection as Internal Control

*E. coli* was detected in all samples tested, indicating efficient nucleic acid extraction and inhibitor removal during sample processing. This finding supports the notion that negative detection of EnV at several sample sites is truly negative, as opposed to being due to unsatisfactory nucleic extraction and/or inhibitor effect.

### PCR Product Sequencing and Analysis

**Figure 3:**
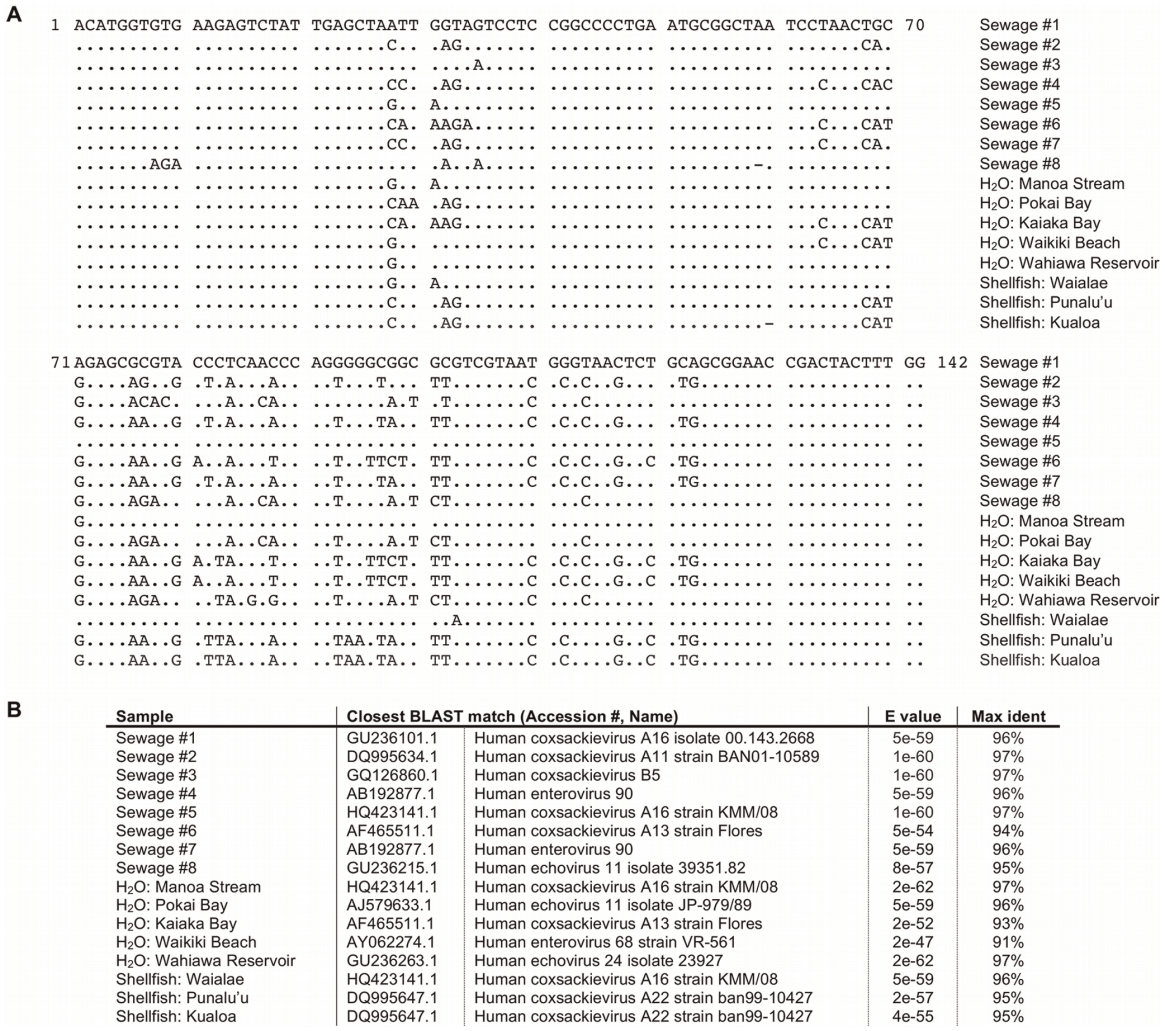
Nucleotide sequence analysis of EnV isolated from wastewater (multiple clones), water, and shellfish samples. (A) Sequence alignment of 142 bp fragments amplified by primer set EQ-1/EQ-2. Dots indicate homology with sewage isolate #1. (B) Closest BLAST match (including E value and percentage identity) of sequenced PCR products with EnV strains listed in the NCBI database.

Sequencing and BLAST analysis from selected EnV-positive sewage, water, and shellfish samples revealed high sequence homology with a variety of EnV strains listed in the NCBI database (Figure 3), as expected when using a primer set broadly reactive for all enterovirus types. Of the 16 sequenced EnV PCR products, 12 were identified as human coxsackie A/B viruses (including human enterovirus 90), causative agents of herpangina, meningitis, fever, respiratory disease, hand-foot-and-mouth disease, myocarditis, heart anomalies, thrush, pleurodynia, and diabetes [36]. Also detected were human enterovirus 68, associated with respiratory illness [37], and 2 human echoviruses, linked to meningitis, fever, respiratory disease, thrush, gastroenteritis, and severe neonatal infections [38].

### Enterovirus Infectivity Assay

Results from the infectivity assay showed no obvious viral-induced CPE in any of the three cell lines exposed to urban wastewater shown to be EnV-positive by RT-PCR, even after one month of incubation and blank passages. Possible explanations are discussed below.

### Biostatistical Analysis

Based on the comparative data in Table 5, a statistical score test reveals significant association between EnV detection in water and shellfish samples (p-value = 0.0410).

## Discussion

Reported here is a rapid, user-friendly method for the effective concentration and detection of enteroviruses from Hawaiian environmental waters. Because reliance on bacterial indicators alone for water quality surveillance fails to reflect the presence of potentially problematic viral pathogens, a need for alternative monitoring parameters exists [2]–[4]. The conveyed method provides a practical means of utilizing enteric viruses as alternative indicators, with potential to enhance accurate assessment of microbial water quality and minimize risks associated with polluted recreational waters. By using urban wastewater as our nucleic acid source for initial protocol establishment, as opposed to a single clinical sample, primer sensitivity was optimized for a broad genotypic range of all human enteroviruses. By comparing detection efficiencies of presently available primer sets in a side-by-side manner, we were able to establish EQ-1/EQ-2 to be a fine-tuned, and highly sensitive protocol for effective enteroviral detection. Under the described conditions, this optimized protocol is 10^3^- to 10^7^-fold more sensitive than all other protocols tested, suggesting its suitability to detect viral pathogens present in water environments at low concentrations. Establishment of this sensitive method allowed a survey study of 22 recreational water sites around the island of Oahu, 11 of which tested positive for enterovirus, indicating fecal pollution in a significant portion of Hawaii’s surface water. It is worthy to note that this is the first report of using an effective molecular detection method to demonstrate a relatively high occurrence of enterovirus in Hawaiian recreational waters.

While molecular detection assays can be useful for indicating fecal contamination in an area, they do not distinguish between the presence of a specific nucleic acid sequence or complete, viable, and infectious virus particles [3]. Therefore, infectivity assays based on the observance of viral-induced CPE in cell culture are important in order to make valid determinations of health risks [6]. The negative result from our infectivity assay could be attributed to various reasons, including: 1) inefficient infection of test cells due to limited viral particles (< an infectious dose) recovered from a relatively small sample volume (100 ml); 2) enteroviruses present in this sewage sample were truncated, non-infectious viral particles, despite positive RT-PCR detection of the EnV genome; 3) other suboptimal aspects of our infectivity assay protocol, such as viral recovery from membrane, culture conditions, etc. Ongoing work in this laboratory is aimed at establishing a more reliable protocol for determining the relationship between enteroviral persistence (detected by molecular methods) and infectivity (determined by CPE-based *in vitro* cell culture method). However, regardless of infectivity results, sensitive and efficient molecular detection of EnV remains a highly valuable resource for indicating current or recent fecal contamination in recreational waters. Even if PCR-detected enteric viruses present in a particular water sample are not directly associated with disease outbreak, their positive detection is indicative of the potential presence of other enteric pathogens of concern.

Of notable practical significance is that comparable optimization studies in our laboratory have produced similar protocols for the efficient environmental detection of other human enteric viruses, including adenovirus [17], norovirus genogroups I and II [39], and fecal coliphage [40]. When combined with the EnV detection protocol established here, these procedures comprise a powerful array for monitoring and comparing fecal pollution levels. The ability to reliably screen environmental waters for the presence of multiple strains of enteric viruses is a highly desirable research tool, facilitating a thorough investigation of potentially contaminated recreational waters. The relatively simple protocols using well-established, conventional RT-PCR procedures are adoptable by a broad range of environmental health agencies, for which more advanced equipment and techniques (e.g. real-time PCR) may be unavailable.

The novel use of shellfish as bioindicators of water quality explored here also has interesting implications for enhanced environmental surveillance. Because these animals process large volumes of water daily through filter feeding, any pollutants present in the water, including viral pathogens, bioaccumulate within the internal tissues of the shellfish [15], [16]. By testing these animals for the presence of enteric viruses, this natural bioconcentration phenomenon may be utilized as a means of assessing microbial water quality. As shown in Table 5, shellfish dissection, nucleic acid extraction, and subsequent PCR analysis revealed positive EnV detection in specimens from six of nine beach locations. Water collected from the three sites where EnV was not detected in shellfish also tested negative; this correlation suggests that these sites are free of EnV contamination. Four of the six sites where EnV was detected in shellfish, including Kahala Beach and the beach parks at Ala Moana, Ko Olina Lagoon 3, and Punalu’u, were also shown to contain EnV through membrane filtration of water samples. This positive correlation strongly suggests fecal pollution at these four beaches. It is interesting to note that from the remaining two sites, Waialae Beach Park and Kualoa Regional Park, where shellfish were shown to contain EnV, water tested EnV-negative. This result suggests that using shellfish as sentinels of water quality is a more sensitive monitoring method than testing water directly. However, while detection efficiency may be increased, this method does require additional processing time and effort, as an adequate number of shellfish must be acquired and dissected prior to nucleic acid extraction. Thus, this method may be more suitable for in-depth water quality studies, while the ease and simplicity of direct water sample collection may be more practical for routine recreational water monitoring. Future research, including laboratory-controlled spike studies to measure bioaccumulation and inhibition levels, will further investigate the practical feasibility of using shellfish as natural and competent bioindicators of water quality.

Although the described methods are powerful supplements to aid microbial water quality monitoring, we realize that without more conclusive infectivity data, public health implications are limited. Risk assessment at any particular recreational site cannot be based solely on PCR-detected EnV presence or absence from a single sample collection. Additionally, our present study is limited to the detection of EnV strains present in Hawaii, which may not be a complete representation of the EnV composition present elsewhere. For serious consideration as a valid and established alternative monitoring system, broader large-scale trials, including additional sampling sites and replicate samples from each site, will be necessary. Also, comparisons with standardized bacterial surveillance systems will contribute to a more thorough understanding of water quality assessment.

In summary, the highly sensitive approaches reported here for EnV detection from recreational waters will be extremely useful tools for environmental virologists and are important stepping-stones, leading toward the concrete establishment of model alternative water quality monitoring systems. Particularly marine shellfish are potentially useful for enhanced detection efficiency of enteric viruses, Although it is currently unknown whether EnV detected in environmental samples by RT-PCR exists as infectious virus particles, positive molecular detection is still a significant indication of fecally-polluted recreational waters. The high enterovirus prevalence detected in Hawaiian waters should heighten awareness of possible fecally-derived waterborne pathogens and instigate additional surveillance of our precious recreational waters.
